# Red Panda feces from Eastern Himalaya as a modern analogue for palaeodietary and palaeoecological analyses

**DOI:** 10.1038/s41598-021-97850-y

**Published:** 2021-09-15

**Authors:** Sadhan K. Basumatary, Rajib Gogoi, Swati Tripathi, Ruby Ghosh, Anil K. Pokharia, H. Gregory McDonald, Norbu Sherpa, Eline N. van Asperen, Rajesh Agnihotri, Geetamani Chhetri, Korobi Saikia, Arya Pandey

**Affiliations:** 1 Birbal Sahni Institute of Palaeosciences, Lucknow, Uttar Pradesh India; 2 Botanical Survey of India, Sikkim Himalaya Regional Centre, Gangtok, Sikkim India; 3 Bureau of Land Management, Colorado State Office, 2850 Youngfield Street, Lakewood, CO 80215 USA; 4 School of History, Classics and Archaeology, Newcastle University, Newcastle upon Tyne, UK; 5 G. B. Pant, National Institute of Himalayan Environment (NIHE), Gangtok, Sikkim India

**Keywords:** Ecology, Evolutionary ecology

## Abstract

Modern feces samples of the endangered red panda (*Ailurus fulgens*) were examined using multiproxy analysis to characterize the dietary patterns in their natural habitat in India. An abundance of Bambusoideae phytoliths and leaves (macrobotanical remains) provide direct evidence of their primary dietary plants. In contrast, Bambusoideae pollen is sporadic or absent in the pollen assemblages. An abundance of *Lepisorus* spores and its leaves along with broadleaved taxa, *Betula*, *Engelhardtia*, and *Quercus* are indicative of other important food sources. Average δ^13^C values (**− **29.6‰) of the red panda feces indicate typical C_3_ type of plants as the primary food source, while the, δ^15^N values vary in narrow range (3.3–5.1‰) but conspicuously reveal a seasonal difference in values most likely due to differing metabolic activities in summer and winter. The multiproxy data can provide a baseline for the reconstruction of the palaeodietary and palaeoecology of extinct herbivores at both regional and global scales.

## Introduction

Arboreal wildlife is extremely sensitive to the deterioration of natural vegetation, climatic change and anthropogenic activities. Ongoing anthropogenic warming has resulted in global climate change which has threatened key wildlife species such as the red panda as well as endangering its habitat. The change in monsoonal conditions has affected the vegetation of the eastern Himalaya, resulting in a high degree of risk for some of listed endangered faunal species such as red panda^1,2^. Due to the negative impact on this species, serious efforts are being taken for conservation at regional as well as global levels. Human efforts can control environmental factors only to a certain extent; therefore, efforts are also being taken to develop climate change resistive/adaptive species to enhance resilience of both flora and fauna in the face of a rapidly changing global ecology. To achieve that, it is critical to understand seasonal dietary patterns, factors that limit a species distribution and metabolic pathways in a comprehensive manner.

The underlying causes for the megafaunal extinction at the end of the Pleistocene are still debated and tend to focus on two primary issues, climatic/environmental change and anthropogenic activities, or some combination of these^3–7^. The current rate of population reduction and potential extinction of herbivores and carnivores in the wild is a major global ecological issue. This current extinction event may exceed the end Pleistocene extinction event as currently about 60% of the large herbivorous animals are threatened with possible extinction^8^. Southeast Asia contains the world’s highest number of threatened mammals^9^, with regional faunas experiencing ongoing range reduction and extinctions driven by human activities^10^.

The detailed examination of an animal’s dung, especially the analysis of herbivore dung is one of the best sources to understand their dietary requirement and habitat preference in relation to existing vegetation and climate of a region. In general, herbivorous animals prefer some plants as their primary food, however; preferences may vary seasonally depending on the availability of these plant species in the region. Like modern dung, fossil dung (coprolite) may serve as a critical source of information for the palaeodietary analysis of extinct species and help to understand the palaeoecology of the region^11,12^. Dung and coprolites, along with stomach/intestinal contents, can be considered as the most direct sources of information on the plant species consumed by both extant and extinct herbivores aside from direct observation on an animal’s feeding behaviour, which may not always be possible for modern species^13^ and of course impossible for extinct species. The palaeodietary reconstruction for an extinct species can provide an idea of how it responded to climate and environmental changes prior to human activity and for extant species how changes in their past habitat influenced their current distribution^14^.

Besides the micro and macrobiological examination of dung, stable carbon and nitrogen isotopic values (δ^13^C and δ^15^N) are powerful tools to evaluate/characterize the ‘ecology based’ diet pattern of a species based on the isotopic difference between C_3_ and C_4_ plants (~ − 27‰ to − 30‰ for C_3_ plants compared to ~  − 14‰ to − 8‰ for C_4_ plants) in an herbivore’s diet. This information helps to establish the species relationship with surrounding vegetation and climate of the region. Earlier studies examining both coprolites and modern dung have enhanced our understanding about the palaeodietary preferences of extinct species from different parts of the world and at different intervals of geological time^13,15–19^.

Some studies have also examined the nutritional elemental content of herbivore dung in relation to the dietary habits of the species and the effect on vegetation composition in arid regions^8,20^. Palynological studies have been carried out on modern dung middens to understand how the food habits of herbivores are related to the existing vegetation of a region^21–23^.

With respect to the red panda, some preliminary works have been carried out on its food plants from India, Bhutan, Nepal, and China including analysis of its feces^24–34^. However, these past studies have only focused on the macrobotanical remains in the feces. The main aim of this study is to provide a multiproxy record on the endangered arboreal herbivore, the red panda to provide more detailed information on the plants in their diet in their natural habitat in the region. The study also provides a baseline to facilitate accurate identification and characterization to distinguish the coprolite of arboreal and terrestrial herbivores in relation to palaeoecological analyses at regional and global levels.

The red panda is currently classified into two phylogenetic species, the-Himalayan red panda (*Ailurus fulgens*) and Chinese red panda (*Ailurus styani*)^35^. The red panda is listed as an endangered species and has been referred to as a living fossil, although its highly derived morphology is far removed from the group’s typical morphology, as reflected by the extreme adaptations of the skull and dentition for a vegetarian diet^36,37^. The red panda is endemic to the Eastern Himalaya^38^ and its distribution is confined to five Asian countries, Nepal, India, Bhutan, northern Myanmar, and China^39^. In India, its distribution includes Sikkim, Darjeeling and Kalimpong districts of West Bengal, Arunachal Pradesh, and Meghalaya^39^.

**Figure 1:**
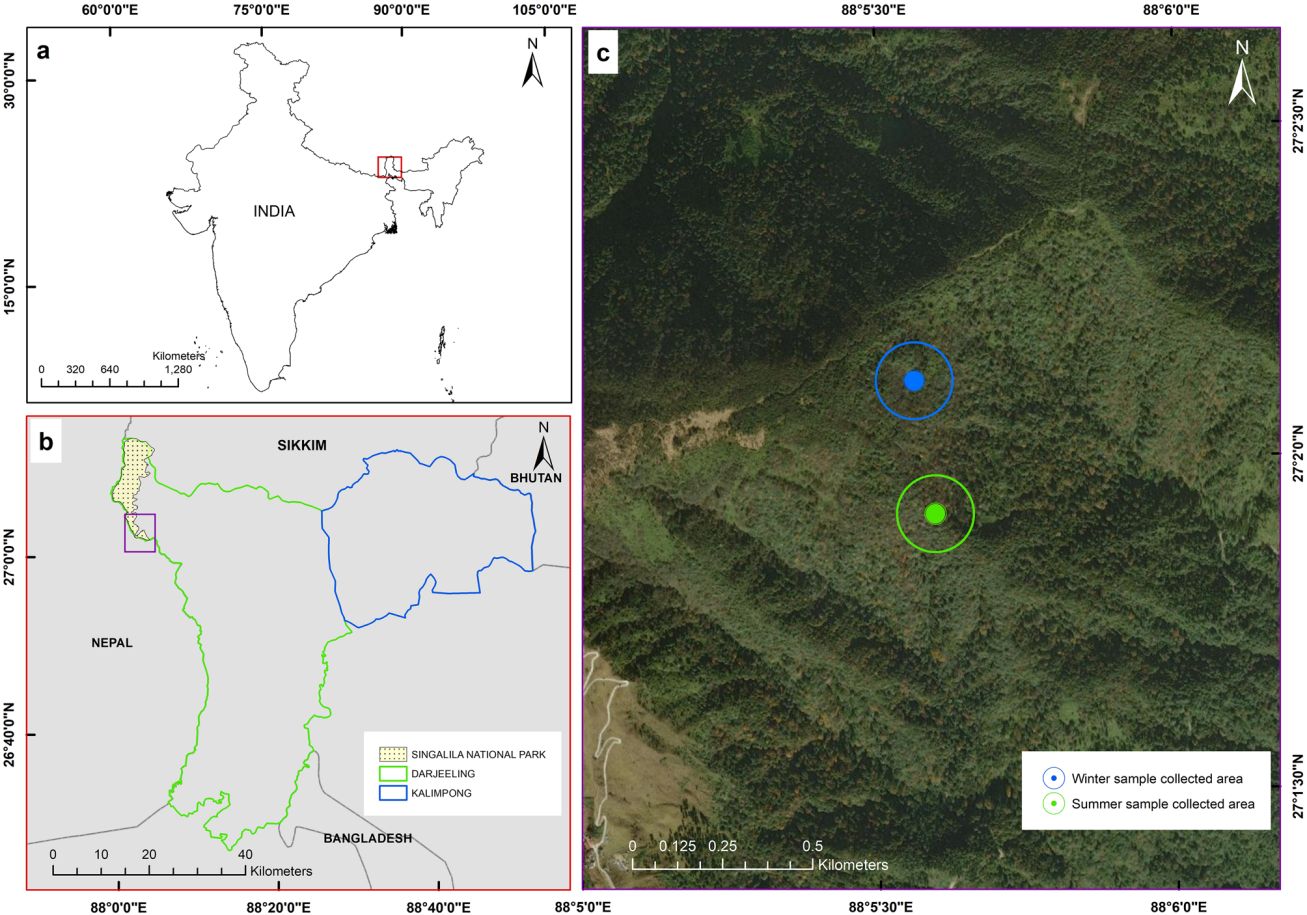
Map compilation showing. (**a)** Darjeeling and Sikkim Himalaya in India (red box), (**b)** outline of Darjeeling Himalaya with Singalila national park, (**c)** expanded view of the sampling site. Map prepared using ArcGIS Desktop 10.5.0.6491 software licensed (2017–2018) to Botanical Survey of India (BSI).

No fossils of either of the living red panda species are known and older reports of fossils that were originally assigned to the genus have since been recognized as other extinct genera. While the red panda, like the giant panda, is a specialist feeder of bamboo, this is the result of dietary convergence as both have distinct phylogenetic positions within the Carnivora^40,41^. The study area included Singalila National Park and its vicinity in the eastern Himalaya (Fig. 1). This area is considered one of the best sites to observe the Himalayan red panda in its natural habitat and has been used for reintroductions of captive bred individuals^42^. The park includes subtropical to sub-alpine zones. In general, the vegetation is composed of sub-alpine coniferous forest with *Rhododendron* scrub, temperate broad-leaved mixed forest with bamboo thickets and subtropical forests (Supplementary Fig. 2a, b). The temperate broad-leaved mixed forest with bamboo thickets is represented by *Acer campbellii*, *Brassaiopsis mitis*, *Quercus pachyphylla*, *Betula utilis*, *Magnolia campbellii*, *Rhododendron arboreum*, *R. grande*, *Osmanthus suavis*, *Rosa sericea*, *Rubus wardii*, and the bamboo species, *Yushania maling*, *Thamnocalamus spathiflorus, Arundinaria racemosa*, and *Himalayacalamus* sp. The sub-alpine coniferous forest is mainly comprised of *Abies densa*, *Tsuga dumosa*, and *Taxus baccata*, along with *Rosa sericea*, *Rhododendron barbatum,* and *R. fulgens* with occasional bamboo thickets of *Thamnocalamus spathiflorus*.

The Himalayan red panda is considered one of the most important wildlife species in the Eastern Himalaya. Other common wildlife include mammalian taxa include *Capricornis thar*, *Naemorhedus goral*, *Muntiacus muntjac*, *Martes flavigula*, and birds such as *Tragopan satyr*, *Cholornis unicolor*, and *Gyps himalayensis*^24^.

The varied topography and range of elevation in Singalila National Park allows for three separate biomes, ranging from subtropical to subalpine. The sub-tropical biome roughly exists between the elevations of 1800–3000 m, and the temperate biome exists in the elevational range of 3000–4500 m^43^. The area experiences a typical monsoon climate with wet summers and dry snowy winters. The region receives heavy rainfall with an average of 3000 mm annually. Temperature ranges from 15 to 20 °C during the summer and the winter temperature is always below 10 °C with snowfall from December–March.

## Results

Modern feces samples of Himalayan red panda were collected and subjected to macrobotanical, pollen, phytolith, stable carbon and nitrogen isotopes and elemental analyses to study their dietary patterns and to established ecological links between the red panda’s diet and regional vegetation as well as to serve as a baseline for palaeoecological studies of the species.

### Macrobotanical remains

Among the macrobotanical remains, bamboo leaves are predominant in the feces showing an average value of 85%, followed by ferns (15%) in summer and 87%, followed by ferns (13%), in winter samples respectively (Supplementary Fig. 2) indicating essentially no seasonal change in the relative importance of these plants in its diet.

### Pollen grains and fern spores

#### *Bambusoideae*-*Betula-Rhododendron-Syzygium-Lepisorus assemblage*

**Figure 2:**
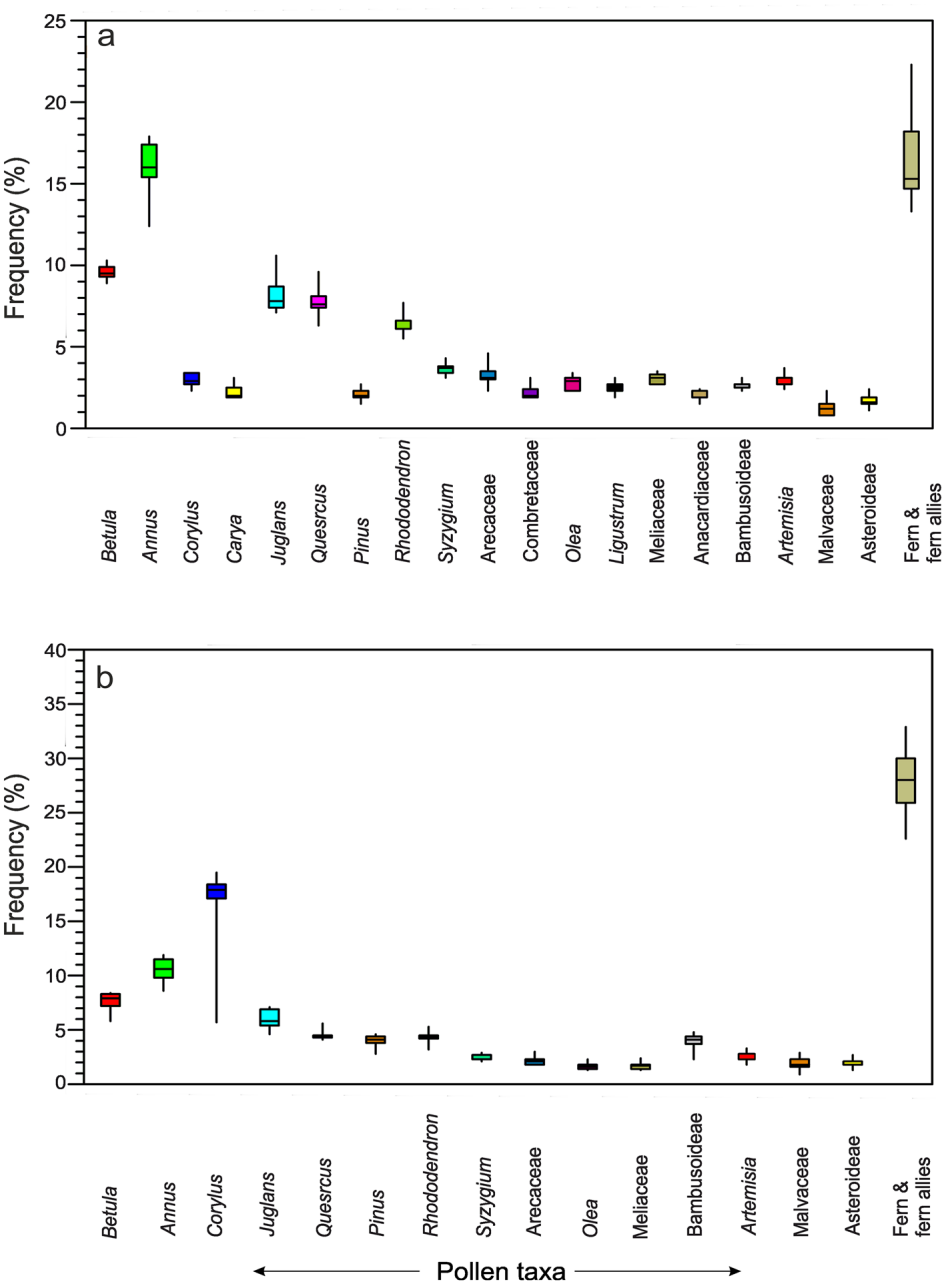
Box plots showing distribution of different pollen taxa in summer (**a)** and winter feces (**b)** samples.

The 9 summer feces samples from the different locations show that arboreal pollen taxa (77.6% of the total pollen count) are dominant followed by ferns (16.4%) and non-arboreal taxa (5.9%). Among the local arboreal taxa, *Betula*, *Quercus*, Arecaceae, *Ligustrum*, and *Rhododendron* are represented in significant amounts and vary between 1.9% and 17.9%. Bambusoidae pollen is found to vary between 2.3 and 3.1%. Among the non-arboreal taxa, *Artemisia*, Malvaceae, and Asteroideae constitute about 0.8–3.7%. Fern and fern allies vary between 4.9 and 11.6% in the palynoassemblages (Fig. 2).

#### *Bambusoideae*-*Juglans-Arecaceae-Rhododendron-Lepisorus assemblage*

In the winter samples the arboreal taxa are also dominant but with reduced frequencies (65.7% of the total pollen count), followed by ferns (27.9%) and non-arboreal taxa (6.4%) respectively. Among the local arboreal taxa, *Betula*, *Engelhardtia, Quercus*, and *Rhododendron* are represented between 1.4 and 19.5%. Bambusoideae pollen is recorded at values between 2.3 and 4.8% in the palynoassemblages. Among the non-arboreal taxa, *Artemisia*, Malvaceae, and Asteroideae are recorded between 0.9 and 3.1%. Ferns and fern allies vary between 6.9 and 20.9% (Fig. 2).

### Detrended component analysis (DCA) and principal component analysis (PCA)

**Figure 3:**
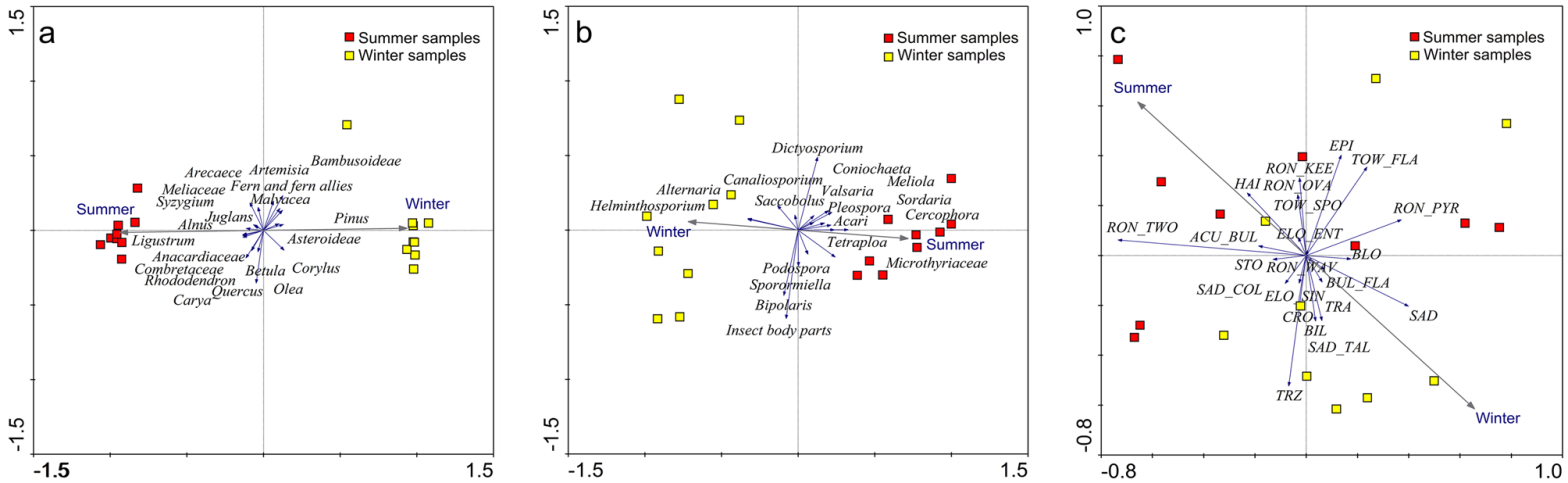
Comparative PCA ordination plots for the (**a)** pollen, (**b)** NPP and (**c)** phytoliths recovered in the summer and winter faeces samples.

The indirect gradient analysis suggests that a long environmental gradient does not exist based on the gradient lengths of the axis (Supplementary Table 1). However, the first axis is the most dominant as exhibited by its higher eigenvalue compared to the other axes. Principal Component Analysis (PCA) ordination analysis also shows that the first axis can explain about 89.0% of the total variations observed in the palynomorph data. It is also apparent in the ordination plot that summer and winter samples vary significantly in their palynomorph compositions. The summer samples show strong correlations with Arecaceae, Anacardiaceae, Combretaceae, *Juglans*, *Ligustrum*, *Quercus*, *Rhododendron*, and *Syzygium*, while Asteroideae, *Corylus*, *Olea,* and *Pinus* are closely associated with the winter samples (Fig. 3).

### Non-pollen palynomorphs

#### Sporormiella-Podospora-Sordaria-Alternaria-Helminthosporium-Acari assemblage

**Figure 4:**
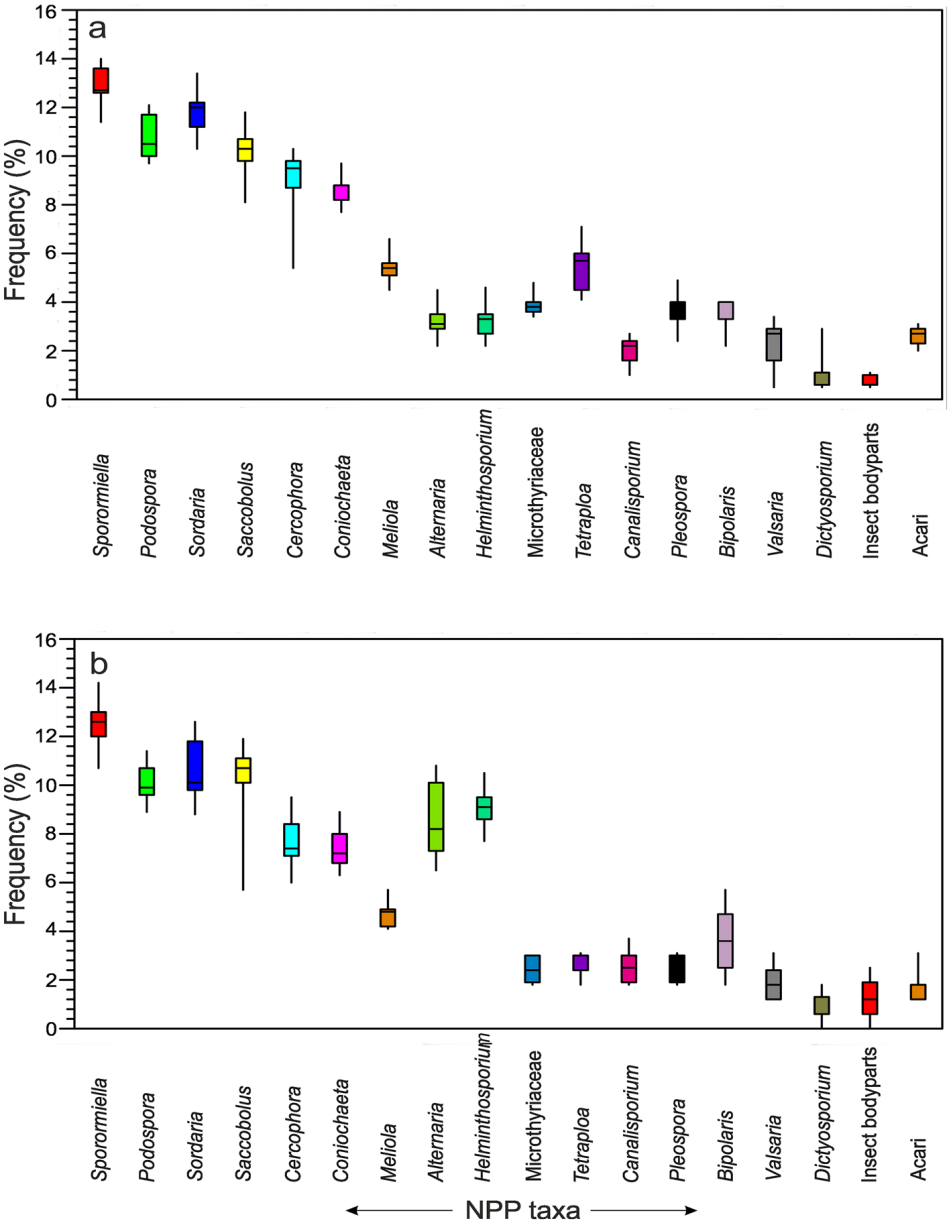
Box plots showing distribution of different NPP taxa in summer (**a)** and winter feces (**b)** samples.

The fungal spores were retrieved from the same palynological slides as the summer feces samples. A dominance of coprophilous fungal spore types was observed (c. 63.0% of the total NPP count) followed by non-coprophilous fungal spore types (34.4%) and Acari (2.6%) in the assemblages. Among the coprophilous fungi, *Sporormiella*-type, *Podospora*-type, *Saccobolus*-type, and *Sordaria*-type are dominant and ranged from 5.4 to 14.0% in the assemblages. Among the non-coprophilous fungi, *Helminthosporium*-type, *Bipolaris*-type, and *Tetraploa*-type are consistently represented with values of 1.0–10.5%. Acari are also consistently present in the assemblage (Fig. 4).

#### Sporormiella-Podospora-Saccobolus-Pleospora-Alternaria-Helminthosporium assemblage

Similarly, fungal spores of winter feces samples show that again the coprophilous fungal spore types are dominant (ca. 58.5% of the total NPP count) followed by non-coprophilous fungal spore types (39.7%) and Acari (1.8%). Among the coprophilous fungi, *Sporormiella*-type, *Podospora*-type, and *Sordaria*-type are dominant and range between 5.7 and 13.0%. Among the non-coprophilous fungi, *Helminthosporium*-type, *Alternaria*-type, and Microthyriaceae vary between 0.7 and 10.8%. Acari are also present in some samples (Fig. 4).

As in the palynological data, Detrended Component Analysis (DCA) shows a gradient length < 2 SD for the first four axes but the higher eigenvalue of axis 1 suggests its dominance (Supplementary Table 1). Results of the PCA show that the first axis can explain about 55.9% variations in the NPP data allowing the summer and winter samples to be successfully distinguished. Coprophilous fungi, including *Podospora*-type, *Sporormiella*-type and *Saccobolus*-type are common in both winter and summer samples, while the *Sordaria*-type is somewhat more associated with the summer feces samples. In contrast, non-coprophilous fungi, *Helminthosporium*-type and *Alternaria*-type, are more closely associated with the winter samples (Fig. 3).

### Diatoms

The number of diatoms in the summer samples was very low and not suitable to make a proper diatom spectrum. No diatoms were observed in the winter samples and *Sellaphora* is the only taxa observed in some summer samples.

### Phytolith assemblages

**Figure 5:**
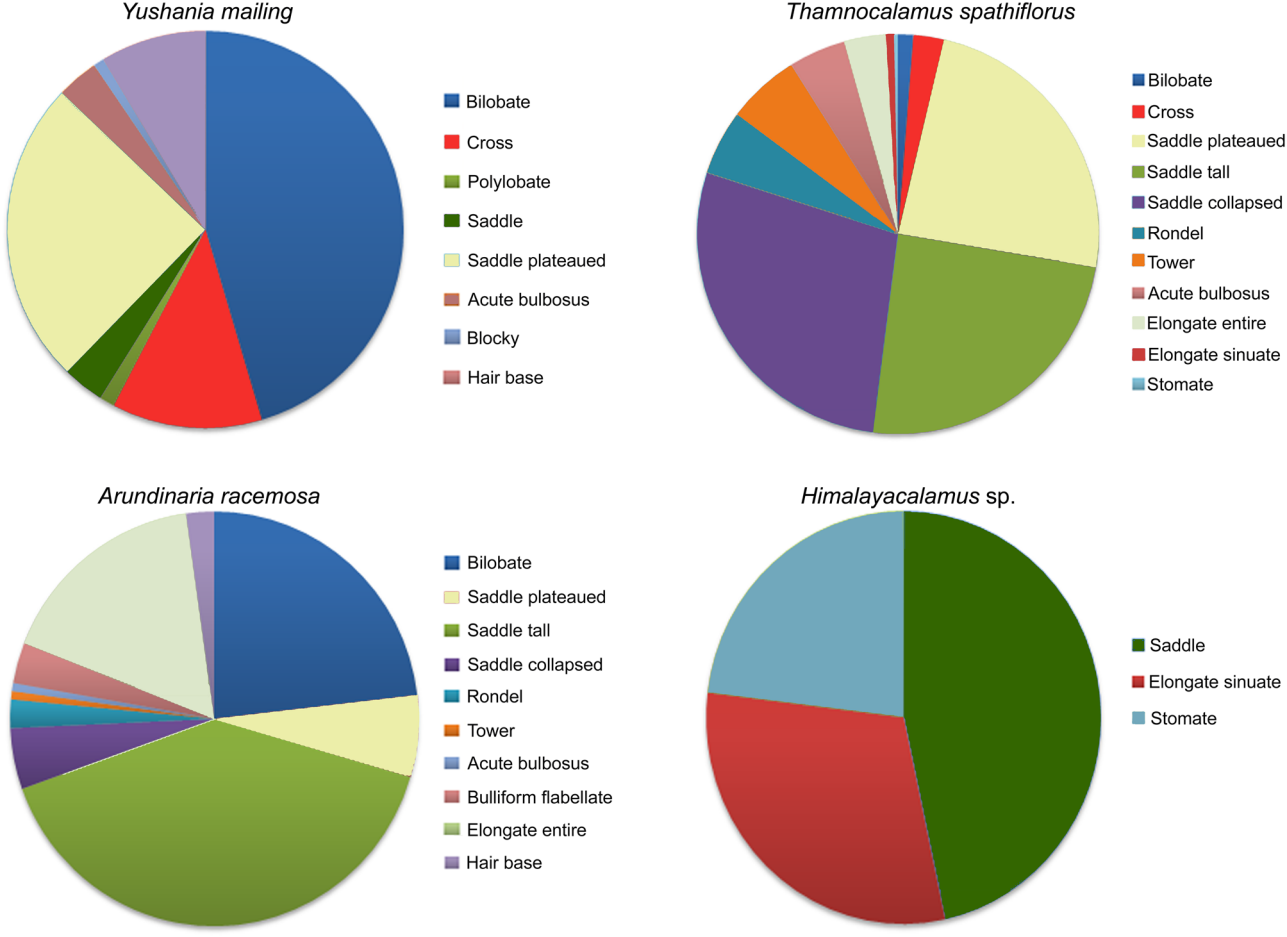
Pie diagrams showing relative abundance of different phytolith types in some dominant Eastern Himalayan bamboo species.

The acronyms used for the phytolith morphotypes from the modern Bambusoideae in the feces of the red panda are provided (Supplementary Table 2). A total number of 22 grass phytolith morphotypes have been recovered. Frequencies of phytolith morphotypes recovered from the modern Bambusoideae are represented (Fig. 5). It has been observed that a wide variability exists in the phytolith production patterns of these Bambusoideae. The feces samples collected during summer and winter seasons show considerable differences in the phytoliths present (Supplementary Table 2).

**Figure 6:**
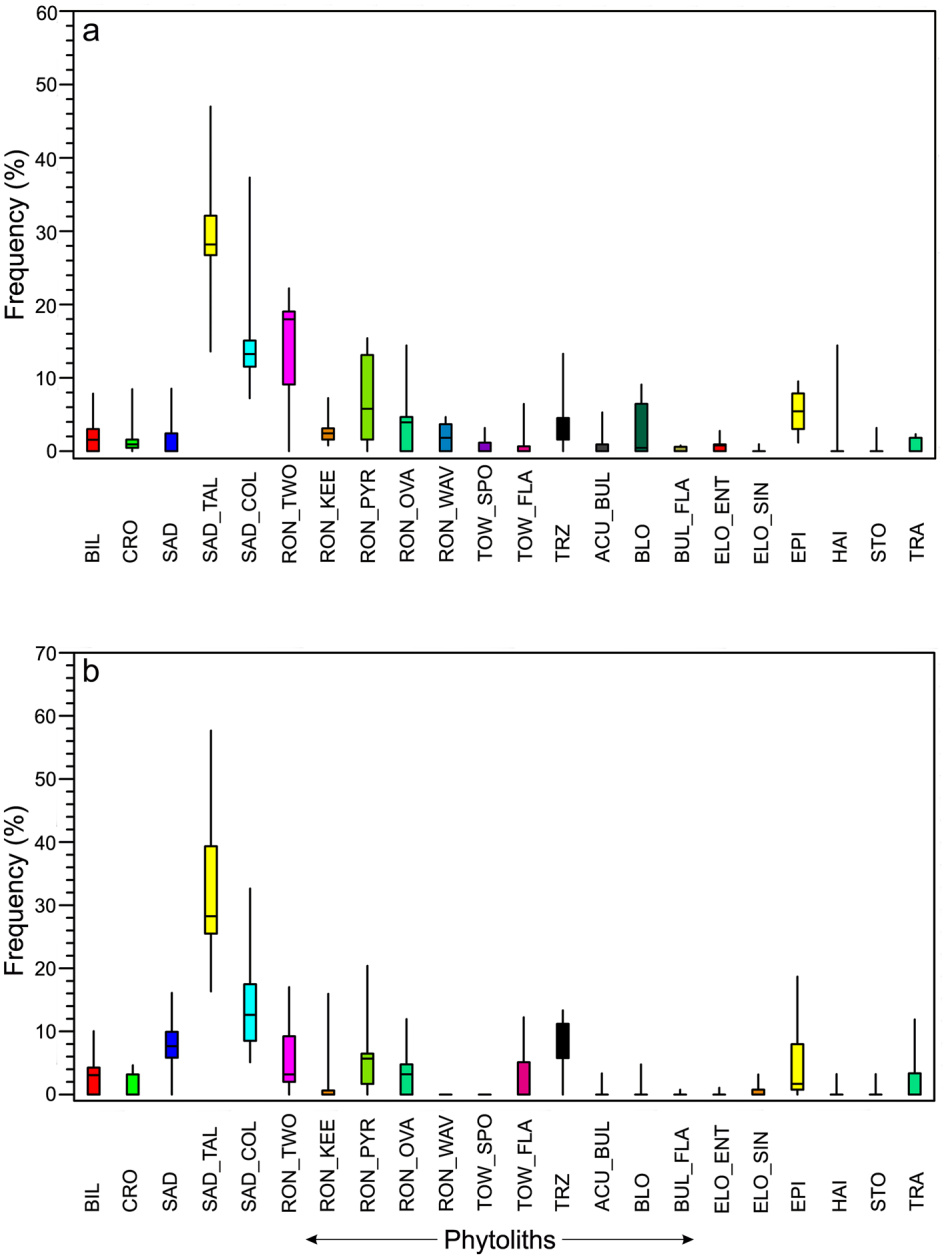
Box plots showing distribution of different grass phytolith types in summer (**a)** and winter feces (**b)** samples.

In the summer samples, SAD_TAL, SAD_COL, and TRZ types are found dominantly. Other rondel (RON_KEE, RON_OVA, and RON_WAV) and tower (i.e., TOW_SPO and TOW_FLA) types show a consistent presence. BIL, CRO, and SAD types also show a consistent presence. Elongate grass phytolith types (ACU_BUL, BLO, ELO_ENT, and TRA) exhibit negligible to moderate occurrences (Fig. 6).

In the feces samples collected during the winter season, BIL, CRO, SAD, and SAD_TAL types show predominance over others. SAD_COL, RON_KEE, RON_PYR, and TRZ have been recovered in comparatively lower frequencies than in the summer samples. Elongated morphotypes show a low to moderate presence (Fig. 6).

As in the pollen and NPP data, DCA results for the phytoliths also show < 2 SD gradient length for the axis, though higher than in the earlier cases. However, from the eigenvalues it is apparent that axis 1 is the most dominant as it shows the highest value. From the results of the PCA it is evident that axis 1 captures about 22% of the total variations in the phytolith data (Supplementary Table 1; Fig. 3). Although summer and winter dung samples could be distinguished using phytolith data there is a minor statistical overlap between the samples. Different RON types and SAD_COL morphs show close associations with the summer samples while BIL, CRO, SAD, and SAD_TAL types show close associations to the winter samples (Fig. 3).

### FESEM-EDS data

A total of 15 major elements were found to be in abundance, characterizing red panda feces samples. The results from the FESEM-EDS elemental analysis of the summer feces samples indicate that the Oxide (O_2_) content is 53.5 (wt. %), followed by C, ~ 36 (wt. %), N, ~ 6.1 (wt. %), Si ~ 2.3 (wt. %), K, ~ 0.6 (wt. %), and Ca, ~ 0.0.4 (wt. %) (Supplementary Fig. 3a; Supplementary Table 3).

Elemental abundance data generated by FESEM-EDS while analysing winter feces samples of Red Panda produced results similar to samples collected during summer. Oxide (O_2_) content/level 54.4 (wt. %) was followed by C, ~ 35.2 (wt. %), N, ~ 5.4 (wt. %), Si, ~ 3.1 (wt. %), K, ~ 0.8 (wt. %), and Ca, ~ 0.4 (wt. %) (Supplementary Fig. 3b; Supplementary Table 4).

### Stable carbon (δ^13^C) and nitrogen (δ^15^N) isotope data

All of the 18 feces samples of red panda were analysed for their C and N concentrations along with their isotopic values. The TC contents vary from 35.5% to 45.1% with an average of 40.8%. The TN contents vary from 1.1 to 2.2% with an average of 1.6%. The δ^13^C values of feces collected during summer closely overlapped (− 29.9‰ ± 0.33) with those collected during winter (− 29.3‰ ± 1.2). This strongly supports the results of the macrobotantical analysis which also indicated a consistent dietary pattern of red panda independent of season.

The δ^15^N values also varied in highly narrow ranges averaging 3.7‰ ± 1.0 and 4.8‰ ± 1.4 for summer and winters respectively, but with a clear indication of enrichment for winter feces samples. The aforesaid trend is obtained when we reject two outlier values of δ^15^N (for samples S-9 and W-16; refer Table 5). It was also noteworthy that average TN contents in summer feces samples were relatively higher (~ 1.8%) compared to those of the winter feces samples (~ 1.5%) (Table 1).

**Table 1:** List of the stable δ^13^C and δ^15^N isotope data in Red Panda feces sample collected from summer and winter season.

Sample No. and ID	IRMS ID	Weight(mg)	TN%	δ^15^N(wrt air)	TC%	δ^13^C (VPDB)	C/N
S1	10,141	3.9753	1.99	3.3	39.38	− 29.7	19.8
S2	10,160	3.3352	2.19	3.3	44.17	− 29.9	20.2
S3	10,144	3.9079	1.77	3.4	39.81	− 29.9	22.5
S4	10,145	3.0934	1.95	3.3	40.20	− 29.8	20.6
S5	10,146	3.8111	1.56	3.3	38.22	− 29.7	24.5
S6	10,156	3.8403	1.93	3.4	36.54	− 30.4	18.9
S7	10,157	4.2286	1.73	3.3	35.48	− 30.1	20.5
S8	10,158	3.2858	1.79	3.3	39.87	− 30.1	22.3
S9	10,104	0.9342	1.3	6.2	45.1	− 29.2	35.1
W1	10,106	3.1777	1.3	3.8	42.5	− 28.8	32.3
W2	10,108	2.4997	1.4	4.1	42.6	− 28.8	30.5
W3	10,110	2.4863	1.2	4.4	42.7	− 28.5	35.5
W4	10,112	1.926	1.1	4.5	41.1	− 28.1	37.4
W5	10,114	2.3486	1.6	4.3	43.5	− 28.4	28.0
W6	10,115	2.7133	1.5	4.0	44.3	− 28.4	30.0
W7	10,161	3.1556	1.56	8.3	39.33	− 30.9	25.1
W8	10,170	4.9328	1.84	5.1	37.89	− 31.1	20.6
W9	10,171	3.8614	1.90	4.5	42.24	− 30.7	22.3

*S1–S9* summer sample; *W1–W9* winter sample.

## Discussion

The multiproxy analysis provides the details of organic and inorganic components in modern feces samples of red panda in relation to their dietary plants and habitat in the region. The micro and macrobotanical remains indicate that members of the Bambusoideae are the primary food resource for the red panda. Our findings revealed a good agreement with the earlier research work pertinent to the red panda diet, where it has been reported that red panda is primarily dependent on bamboo for their survival, though it also consumes other secondary plants growing in their habitat areas^28–30^ .

This is not supported by the limited presence of Bambusoideae pollen which is either absent or only sporadically present in the pollen assemblage. Although bamboo dominated habitat is utilized extensively by the red pandas, this is not reflected in the limited presence of Bambusoideae pollen in the feces which is either absent or only sporadically present in the pollen assemblage. Consequently, in fossil dung, it would not be possible to infer that the primary habitat for this species consisted of bamboo. This absence of bamboo pollen is not unexpected since bamboos only flower at 40–100 year intervals and die after flowering^44^. Therefore, a high presence of bamboo pollen in the feces of the red panda would be a rare occurrence. In marked contrast, broad leaved pollen taxa such as *Quercus*, *Betula*, and *Engelhardtia* are consistently represented in the pollen assemblage at higher values and can provide information on the habitat in which the red panda is found such as the sub-tropical to temperate broad leaf forests and may also be indicative of alternative food plants in the red panda’s diet. The pollen clumping of taxa such as *Rhododendron* and *Betula* is very common indicating a source that is of local origin. The phenology, especially the flowering period of the main tree taxa, was also observed in relation to the representation of the pollen taxa in the pollen assemblage (Supplementary Table 5).

The regular presence of *Artemisia* pollen in the pollen assemblage was marked. Its presence is indicative of areas of open land near the study area. *Artemisia* contains rich mineral nutrients especially calcium and phosphorous^45^ and red panda could be obtaining these minerals from these plants. The presence of *Syzygium* pollen in the pollen assemblage is strongly indicative of the red panda feeding near the perennial river and streamlet system in response to the high rainfall activity in the region. The presence of a perennial water system is an important part of the red panda’s habitat as indicated in the pollen assemblage.

Bambusoideae leaves are hairy and thus effective in trapping pollen and NPP. The presence of *Pinus* pollen, especially in the winter samples of the pollen assemblages, was marked but most likely is indicative of the wind activity in the region and secondary incorporation of pine pollen in the dung, probably through entrapment on the ingested bamboo leaves. Moreover, the consistent presence of fern spores, both monolete and trilete, in the pollen assemblage is indicative of the importance of ferns as food plants and reflects the moist condition resulting from the high rainfall in the region. Consequently, the pollen data recorded from the feces samples of red panda is informative as it reflects the local vegetation and climate in the region. In comparison, the abundance and diversity of the pollen taxa is comparatively higher in the summer than the winter samples where the flowering period occurs mainly in the summer season. This pattern parallels the pollen data recovered from dung of other megaherbivores like wild yak, greater one horned rhino and deer dung samples from Western Himalaya and Eastern Himalayan foothills^22,23,46,47^, directly reflecting the seasonal differences in the local and regional vegetation and climate of the region.

The NPP assemblage, especially the coprophilous fungal spores, chiefly the *Sporormiella*-*Sordaria*-*Podospora-Saccobolus* assemblage, was marked in the studied fungal spore assemblage. After decomposition of the feces these spores can become preserved in the soil and serve as a proxy indicator of the presence of herbivores in the region. This characteristic assemblage in the feces of the red panda is similar to previously recorded fungal remains in other large herbivore dung samples from tropical and temperate parts of India^22,46^ . The recovery of other non-coprophilous fungal spores such as *Alternaria*-type and *Helminthosporium*-type (pathogenic fungi of grass) as part of the fungal spore assemblage was also marked and probably the result of ingesting infected bamboo and other plants. These two types were particularly common in the winter feces, possibly due to feeding on lesser quality food sources. Other fungal spores such as *Bipolaris*-type (another plant pathogen), *Tetraploa*-type, and *Canalisporium*-type are also consistently present in the assemblage. *Tetraploa* is known from a wide range of host plants on leaf bases or stems just above the soil. *Canalisporium* species are common saprotrophs in the tropics and are often found on submerged wood^48^. Along with *Pleospora*, which also favours wet environments, this genus is indicative of the humid climatic condition in the region. A variety of endophytic and saprotrophic taxa (e.g. *Meliola*, *Valsaria*, and *Dictyosporium*) are also present.

Acari, although poor in terms of their indicator value, are generally encountered in herbivore dung, which is helpful for the characterization of modern dung and coprolite analysis. The recovery of insect body parts in the assemblage was also recorded which is indicative of their importance as a food source supplying protein. Though the diatom assemblage is poor, the presence of *Sellaphora* in some samples is indicative of a perennial water-logged and streamlet system from which this diatom would have been ingested through the drinking of water. *Sellaphora* generally occurs in fresh water in both low and high mountainous lakes of glacial origin and prefers the acidic environment in the region^49^.

Seasonal variations in the dietary preferences of the red panda are also supported by the phytolith data. Based on the analysis of the modern Bambusoideae phytolith it is apparent that there is wide variability in the phytolith production patterns within the Bambusoideae. No ‘diagnostic morphotype’ or ‘assemblages’ could be ascertained for this sub-family^50,51^ due to the high diversity and redundancy of the morphotypes present in the dung samples. The redundancy of grass phytolith types among different taxa also limits the identification of the most preferred bamboo taxa in the diet of the red panda. The bamboos, *Y*. *maling*, *A*. *racemosa*, and *T*. *spathiflorus*, grow within the sub-tropical/sub-alpine zones^52,53^ while *Himalayacalamus* sp. is present in the tropical and upper-temperate forests. The phytoliths of these Bambusoideae also show considerable variations. For example, the SAD type is only observed in *Y. maling* and *Himalayacalamus* sp. However, in *Himalayacalamus* sp., the SAD type is more predominant than in *Y. maling* (Fig. 3).

It is worth mentioning that in the Himalayan grasses BIL, CRO, SAD, SAD_TAL, SAD_COL, TRZ, and RON types show significant elevational trends^50,51^. The BIL, CRO, and SAD types are predominant in the grasses of the tropical and sub-tropical zones, while the TRZ and RON types are dominant within the grasses of the sub-tropical/temperate zone and upwards. Phytolith types such as SAD_TAL and SAD_COL are most dominant in temperate zones but decline at higher elevations^50,51^. A seasonal trend has also been also noticed for these phytolith types retrieved in the feces samples. In winter samples they show a high abundance of BIL, CRO and SAD types suggesting that the red panda might have foraged on the bamboo species present at comparatively lower elevations in sub-tropical/lower temperate sites. In contrast, there is a predominance of RON and TRZ types in summer samples indicative of the ingestion of bamboos from comparatively higher elevations of the upper-temperate/sub-alpine forests. These differences suggest a seasonal migration pattern from higher elevations in the summer to lower elevations in the winter, perhaps to cope up with the harsher winter weather.

The FESEM-EDS elemental analysis of both from summer and winter feces was conducted to understand the nutrient elements contained in red panda feces in relation to their dietary plants and climate in the region (Supplementary Table 3 and4; Supplementary Fig. 3). The value of nitrogen content in the N: P ratio (49.0 and 61.3 weight %) in the red panda’s feces is indicative of the arboreal nature of their diet. Nitrogen rich dung is characteristic of a mixed-feeder or browser^20^. Both the winter and summer elemental contents are closely similar indicating there was no decline in the elements in the red panda diet during the year and is consistent with the macrobotanical and stable isotope data that its dietary plant preference does not change with the seasons. These major elements contained in the red panda feces samples could be useful to understand the nutritional value for their survival in the region and what may be a limiting factor for the presence of red panda in other regions. The recorded data also provides a framework for the differentiation between arboreal and terrestrial herbivores and their conservation in relation to the vegetation composition and forms of land-use at the regional and global level.

The average stable carbon isotope values of red panda feces in both the summer and winter feces samples are − 29.6‰ ± 0.9, which indicates that C_3_ plants are the primary food source of red panda. C_3_ plants in the region are mainly represented by arboreal angiosperms and ferns. However, members of the Bambusoideae are also C_3_ plants and display a range of carbon isotope values between − 26.4‰ and − 30.8‰^54^, which closely overlaps with our data. Other angiosperms and ferns may also have contributed to these values. The dung is a metabolic waste product of the originally consumed food^55^. The average TN content in summer dung samples are relatively high (~ 1.8%) as compared to that of winter dung samples (~ 1.5%). The average δ^15^N value of summer feces samples of red panda is highly consistent and relatively lowers (3.7‰ ± 1.0) as compared to winter dung samples (4.8‰ ± 1.4). These summer and winter difference of δ^15^N isotope values could be related to the different rates of metabolic activity in the red panda. During summer, the metabolic activity of red panda is expected to be relatively faster as compared to that of winter resulting into the different fractionations of nitrogen isotopes between the two seasons. The δ^13^C values of red panda feces appear to be independent of metabolic rates (falling within a very narrow range) and controlled by source end-member (food). Effects of environmental factors such as humidity, light and canopy coverage effect should be considered for in future studies^56–58^.

Taken together, narrow ranged δ^13^C and δ^15^N values presented here could be potentially used for the interpretation of the palaeoecology and palaeodietary analysis of extinct species especially determining possibly arboreal or terrestrial habits in herbivores. These data are also helpful for the identification of browsers and grazers where both types of animals were present^59,60^. With respect to the evolutionary history of the Ailuridae, the data provided here provides a basis for examining the diverse dietary habits of the different taxa based currently only on morphological criteria and determining the evolutionary stages resulting in the highly specialized and restricted diet of the red panda.

The red panda feces samples indicate that levels of arboreal pollen taxa in pollen assemblages within the ranges of 66–78% can be considered representative for an arboreal herbivore. The abundance of macrobotanical remains especially Bambusoideae and *Lepisorus* sp. (epiphytic ferns) leaves with sorus are a strong indicator of arboreal habitats. In contrast, in the dung of terrestrial herbivorous mammals in this region, such as the greater one-horned rhinoceros (*Rhinoceros unicornis*), pollen of non-arboreal taxa predominates with average values of 75%, which may include high values of pollen of Poaceae (31%) along with pollen associated with marshy and aquatic taxa (10%). Similar values have been documented in the dung of the wild yak (*Bos mutus*) with average values of 83% non-arboreal pollen taxa including 45% pollen of Poaceae in the pollen assemblage^22,23,46^. Based on these data it may be possible to successfully differentiate between an arboreal and non-arboreal dietary habit in a mammalian herbivore dung or coprolite as part of determining the palaeodietary and palaeoecology of the species.

*Sporormiella*, is often associated with herbivores, and in the fossil record changes in its abundance in pollen profiles is used as a proxy for the population size of megafauna in the region or their absence^61–64^. As the red panda is an arboreal animal that preferentially defecates in trees, the dung fungi from their dung may not contribute significantly to the soil dung fungal spore archive. However, the position of the dung in trees may facilitate dung fungal spore dispersal to a wider area than dung located on the ground, where dispersal distances generally do not exceed 1 m^65–67^, as well as dispersal to a wider range of herbivore species, such as browsers, which feed mainly at higher vegetation levels. The pollen data recorded from megaherbivore dung is a reliable and complementary dataset to surface soil samples at the regional level^23,46^. In this study also, the generated palynodata demonstrates a good agreement with the current vegetation and climate in the region.

## Conclusions

This multiproxy study provides a method to determine a mammalian herbivore’s preferred dietary plants and the ecology of its habitat. The micro and macrobotanical remains in the feces of the red panda indicate that its diet in the Eastern Himalaya was dominated primarily by bamboo. Other plants such as *Betula*, *Engelhardtia*, *Quercus*, and *Lepisorus*, were also identified as important food plants in their diet.

Documenting the food plants consumed by a species in the wild by direct observation in the field is not always possible, consequently other means of documenting a species' diet are critical. The multiproxy approach to study the feces of the red panda presented here provides a good alternative. This approach of utilizing the feces of an extant species has broader implications as it can also be used to distinguish the habits of extinct species as well and provide a means of generating insights into their palaeoecology such has whether they utilized arboreal or terrestrial open habitats as well as provide information on their palaeodiet, as either browser or grazer. In addition to the micro and macrobotanical remains, the use of stable nitrogen and carbon isotope and elemental data can also aid in an accurate understanding of the dietary habits (arboreal and terrestrial) and habitat preference. The isotope and elemental analysis are also very helpful to understand the nutrient requirements necessary for the individual’s health and the species survival which will be needed in further conservation at both the regional and global level.

The pollen data in the feces is indicative of the species habitat, temperate broad-leaved forest with extensive patches of bamboo forest, in response to the high rainfall activity. Despite the red panda’s dietary preference for bamboo, while pollen is well-preserved in the feces, compared to other plants the pollen of bamboo is not well-represented reflecting the ecology of bamboo in which it only flowers once after a long interval of growth and then dies. The phytolith diversity suggests a seasonal migration pattern of red panda from higher elevations to lower elevations which because of high snowfall/intense cold in the Himalayas during winter.

## Material and methods

### Field work

Direct observation of red panda in their natural habitat in the Eastern Himalaya to determine their dietary preferences and ecology is often difficult due to their small population size, shy and elusive nature and their preferred habitat in remote mountainous terrain.

The permission of the field work has been taken by the Forest Department, Government of West Bengal (India) for the survey of flora and collection of animal feces from the Singalila National Park and vicinity. The collection of the feces of red panda is a challenging task as they generally deposit their excreta on moss covered tree branches and only rarely defecate on the ground (Supplementary Fig. 4a, b). During 2019–2020, two of the authors (RAG, NS) familiar with the flora and fauna of Singalila National Park collected 18 fresh (7–10 days old) feces samples based on their size and shape and surveyed the vegetation composition and climate of the study area (Supplementary Fig. 1a–d). Out of 18 feces samples, 9 samples were collected during the summer (April to August) and 9 were collected during the winter season (October to February). Each dung sample was collected at an interval of 10–15 days to cover the detailed dietary analysis of red panda. Around 7–12 feces pellets were observed in a midden and collected from different locations of the Singalila National Park and vicinity. The samples were selectively collected only from inferred adults based on the feces pellet size. The collected feces were packed separately in ziplock polythene bags to avoid contamination before laboratory processing.

### Laboratory work

#### Morphological study

A total of 18 feces samples (combining 4–5 pellets in a sample) were observed to determine the details of variation in shape and size. The feces are cylindrical in shape and size ranged between 1.5 and 2.0 cm in length and 1.2–1.5 cm in diameter (Supplementary Fig. 4c, d).

#### Macrobotanical analysis

For the macrobotanical analysis, we removed approximately 25 g of feces from 18 samples. The samples were gently boiled in 200 ml 5% KOH solution. After boiling, the material was sieved through a 150 µm mesh and study the remains that were in the sieve (i.e., the fraction > 150 micron). The material was washed 2 to 4 times with distilled water and observed systematically under a Stereobinocular (Leica Z6APO) microscope, and photographs were taken with a Leica DFC295 camera. Identifications of the recovered plant fragments were made through the consultation of the Botanical Survey of India (BSI), Sikkim (India) and relevant published literature (Supplementary Fig. 2).

#### Microbotanical analysis

##### Pollen and non-pollen palynomorph (NPP) analysis

A total of 18 dry/semi-dry feces samples (20 g each) were processed for pollen and non-pollen palynomorphs (NPP) using the standard acetolysis method^68^. Samples were treated with 10% aqueous potassium hydroxide (KOH) solution to deflocculate the sediments, 40% hydrofluoric acid (HF) to dissolve silica, and acetolysis (9:1 anhydrous acetic anhydrite to concentrated sulphuric acid, H_2_SO_4_) for the removal of cellulose. After that, the samples were treated twice with glacial acetic acid (GAA) and washed 3 or 4 times with distilled water. The samples were then transferred to a 50% glycerol solution with a few drops of phenol to protect them against microbial decomposition. Excluding the NPP, 210–263 pollen grains and fern spores were counted from each sample to produce the box plot of pollen taxa. For the identification of pollen grains and ferns, we consulted the reference slides kept in the Birbal Sahni Institute of Palaeosciences Lucknow (India) as well as published papers and photographs^69^ (Supplementary Fig. 5).

Similarly, excluding pollen grains and fern spores, 159–201 non-pollen palynomorphs (fungal spores) along with zoological remains were counted from the same palynological slides and make a box plot. The recovered fungal spores were categorized as coprophilous and non-coprophilous fungi. For the identification of fungal spores, we consulted the published literature and microphotographs^70,71^ (Supplementary Fig. 6).

##### Diatom analysis

For the diatom analysis, 20 g of each sample were treated with concentrated hydrochloric acid (HCl) to dissolve carbonates, if any, and then treated with a mixture of nitric acid (HNO_3_) and potassium dichromate to dissolve the organic content and preserve only the siliceous matter. The samples were then washed with distilled water and permanently mounted on slides with Canada balsam for microscopic observation.

##### Phytolith analysis

For phytolith extraction, the same 18 feces samples were treated in 10% HCl to dissolve carbonates (if any) and then washed thoroughly with distilled water and the supernatant was decanted. Later, the residue was boiled in HNO_3_ to completely remove the organic matter within the faeces and preserve only the phytoliths. Heavy liquid floatation using CdI2 + KI (maintaining the specific gravity at 2.3) was done to float only the phytoliths and the supernatant was retained. Then the supernatant was washed in distilled water and the recovered phytoliths were stored in water. Slides were prepared using polyvinyl alcohol and observed with a compound binocular light microscope. At least 200 phytoliths were counted from every sample.

To determine if there were any seasonal dietary preferences of red panda, and which of the major Bambusoideae species may be their primary dietary resource, we considered phytolith production patterns of the four dominant Bambusoideae from the sub-tropical to sub-alpine zones (between 1500 and 3700 m a.s.l.) of the Darjeeling and Sikkim Himalaya^52,53^ i.e., *Yushania maling*, *Thamnocalamus spathiflorus, Arundinaria racemosa*, and *Himalayacalamus* sp. The phytolith production patterns of *Y*. *maling, T. spathiflorus*, and *A. racemosa* have been considered in previous publications on the modern grass phytolith spectra from the eastern Himalaya^50,51^ .. However, the phytolith production pattern of *Himalayacalamus* sp. has been assessed only in this study. For extraction of the phytoliths from *Himalayacalamus* sp., we adopted a method^42^ that involves washing the plant tissues to remove the superficial debris followed by drying the cleaned tissue at 50–60 °C in a hot air oven and oxidation with concentrated HNO_3_ + NaClO_3_ (in 3:1 ratio) in a hot water bath and washing with distilled water. The residual sediments containing phytoliths were then stored in a vialin water.

Observation and microphotographs were done using an Olympus BX-61 microscope with DP-25 digital camera under 40 × magnifications. Phytolith morphs are identified following the International Code of Phytolith Nomenclature (ICPN2.0) ICPT^72^. (Supplementary Fig. 7).

### Statistical analysis

To determine the key patterns of variations of pollen, NPP, and phytolith assemblages retrieved from the feces samples collected during the summer and winter seasons, detrended correspondence analysis (DCA) was performed^73^. The abundance data of the proxies used detrending by linear segments and down-weighting of rare species. This analysis provides the compositional gradient lengths along the first few DCA axes and helps us to assess if there was any environmental influence on the distribution of these biotic proxies in the dung samples. A gradient length of the first axis > 2 SD (standard deviation) indicates that the environmental gradient is long and suggests using a unimodal based method to understand biotic proxy-environment relationships. In contrast, gradient lengths < 2 SD suggest for a selection of a linear based method to understand the proxy-environment relationships. As one of the aims of this study is to test how feces of red panda provide information about their diet, and if their dietary preference shows any seasonal variations, we confined our study to the indirect gradient analyses. Principal component analysis (PCA) has also been performed to portray the biotic proxy data and the variations of the biotic proxies among the sampling sites. All ordination analyses have been performed in CANOCO version 4.5^74–76^.

### FESEM-EDS analysis

Field Emission Scanning Electron Microscope (FESEM) with Energy Dispersive Spectroscopy (EDS) analysis was also performed using FESEM (JEOL, JSM-7610F) equipped with EDS (EDAX, USA instrument) operated at 25 keV to determine the nutrient elemental composition of the red panda feces in summer and winter respectively (Supplementary Table 3 and 4).

### Stable carbon (δ^13^C) and nitrogen (δ^15^N) isotope analysis

The concentration of carbon and nitrogen along with stable isotopic ratio of red panda feces were performed using stable isotope mass-spectrometer (IRMS) connected with an elemental Analyser (EA) in continuous flow mode at Birbal Sahni Institute of Palaeosciences^77^ (Table 1).

## Supplementary Information

Supplementary Information.
