# Supplementary material for: Red Panda feces from Eastern Himalaya as a modern analogue for palaeodietary and palaeoecological analyses
Source: Sci Rep. 2021 Sep 15;11:18312. doi: 10.1038/s41598-021-97850-y (PMC8443643; doi:10.1038/s41598-021-97850-y)
Supplement: Supplementary file 1 — Supplementary Information. [file 41598_2021_97850_MOESM1_ESM.pdf]

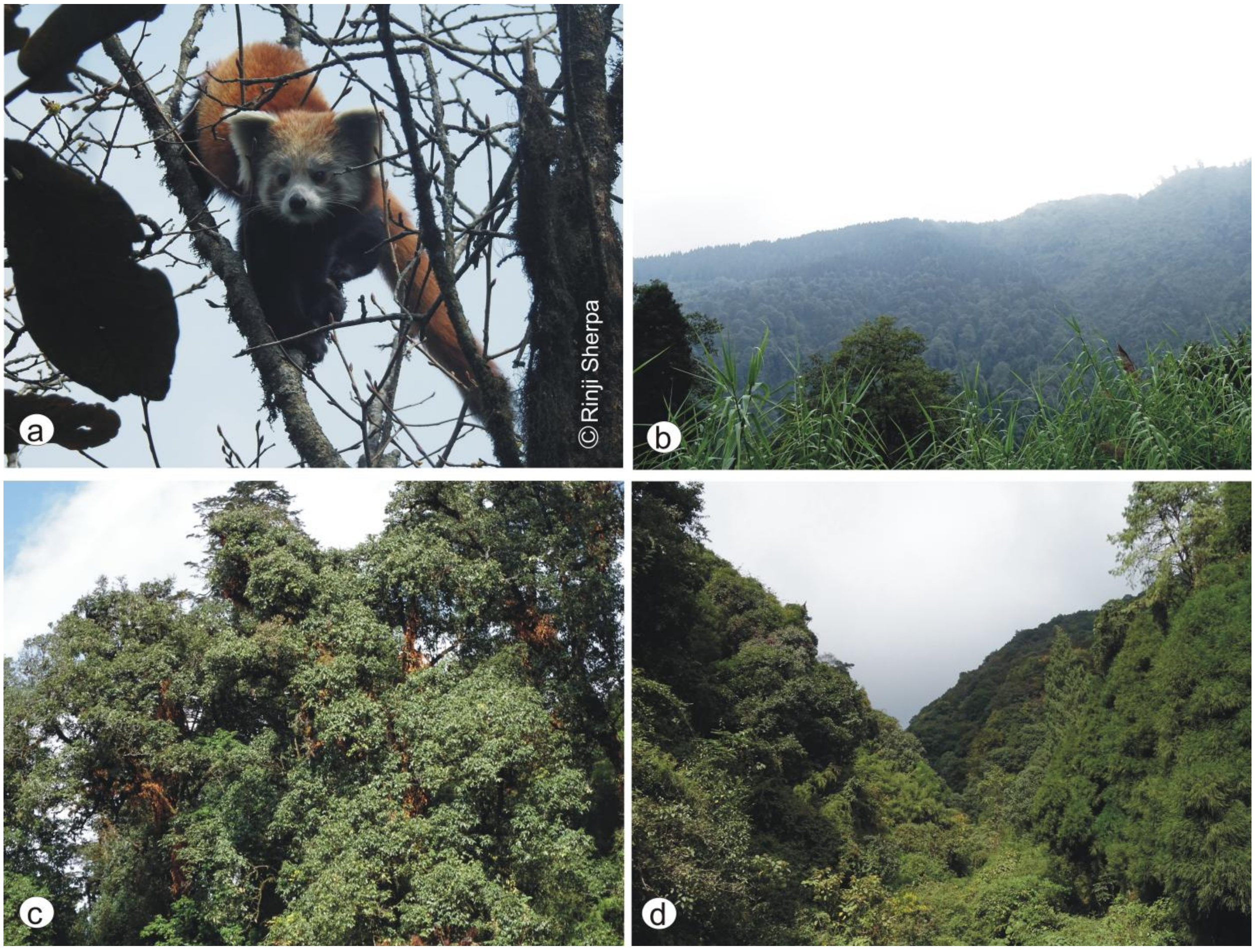

Supplementary Fig. 1. Field photographs, **a.** Red panda in natural habitat (Photo credit: Rinji Sherpa), **b.** A view of forest vegetation in the vicinity of Singalila National Park, **c.** Tree covered with epiphytic fern (*Oleandra* spp.) where red panda prefer to shelter in camouflage, **d.** A view of temperate forest in Singalila National Park.

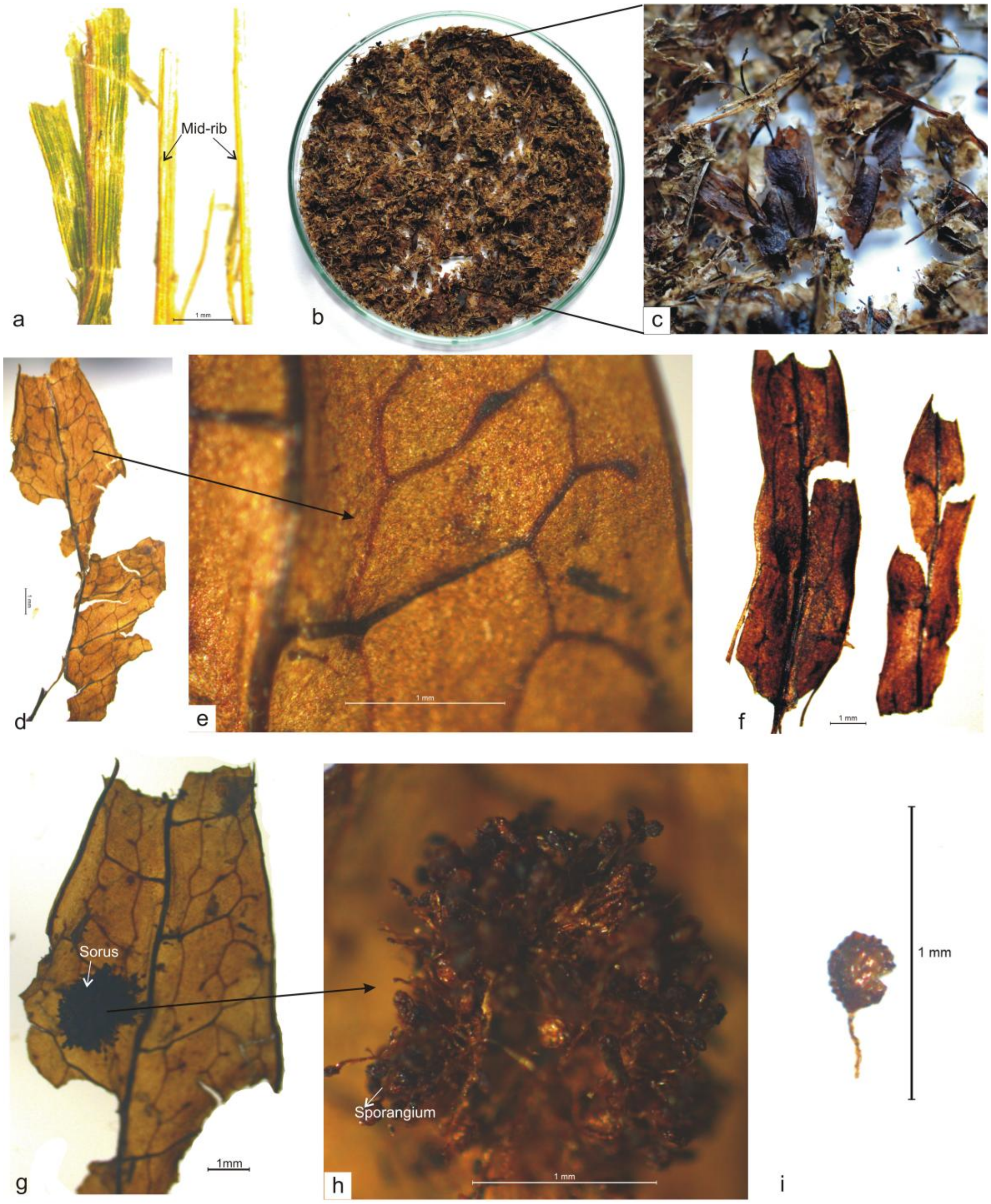

Supplementary Fig. 2. Macrobotanical remains assemblage recovered from the red panda feces.

a. Remnants of undigested bamboo leaf and mid-rib, **b.** *Lepisorus* sp. (Fern) frond, **c.** Enlarged view of fern frond showing dichotomous venation, **d.** Fern frond showing attached sorus on ventral side, **e.** Enlarged view of sorus showing attached sporangium, **f.** Single sporangium.

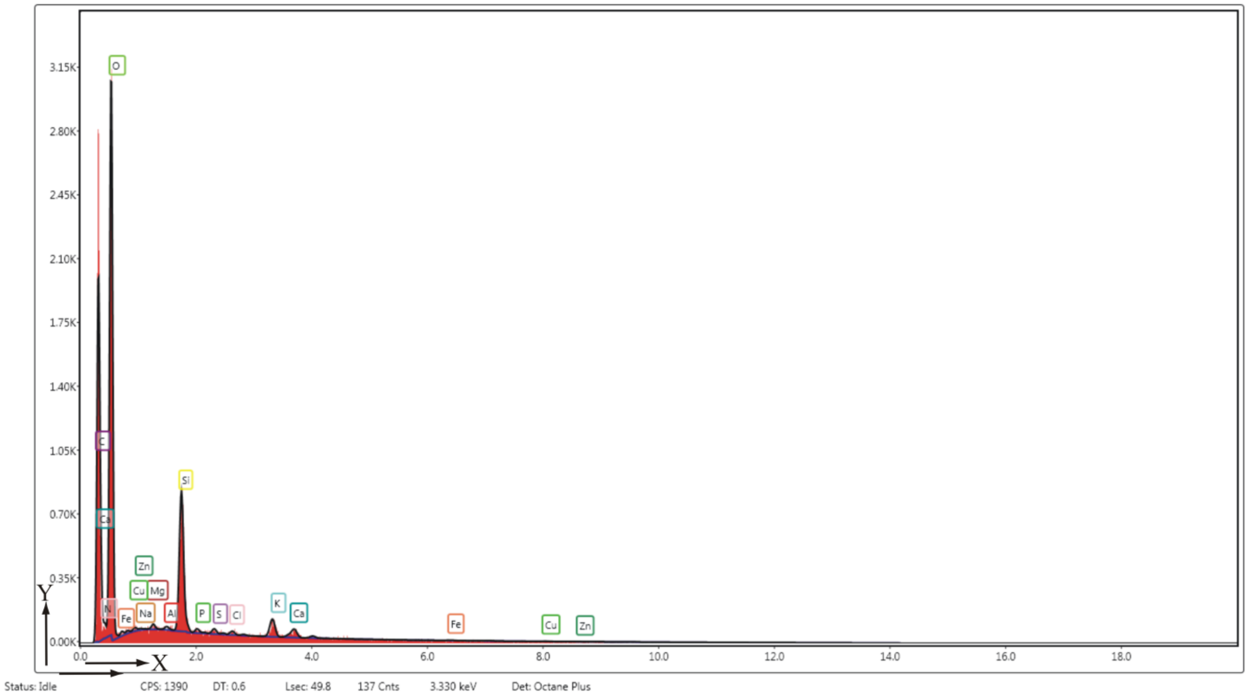

(a)

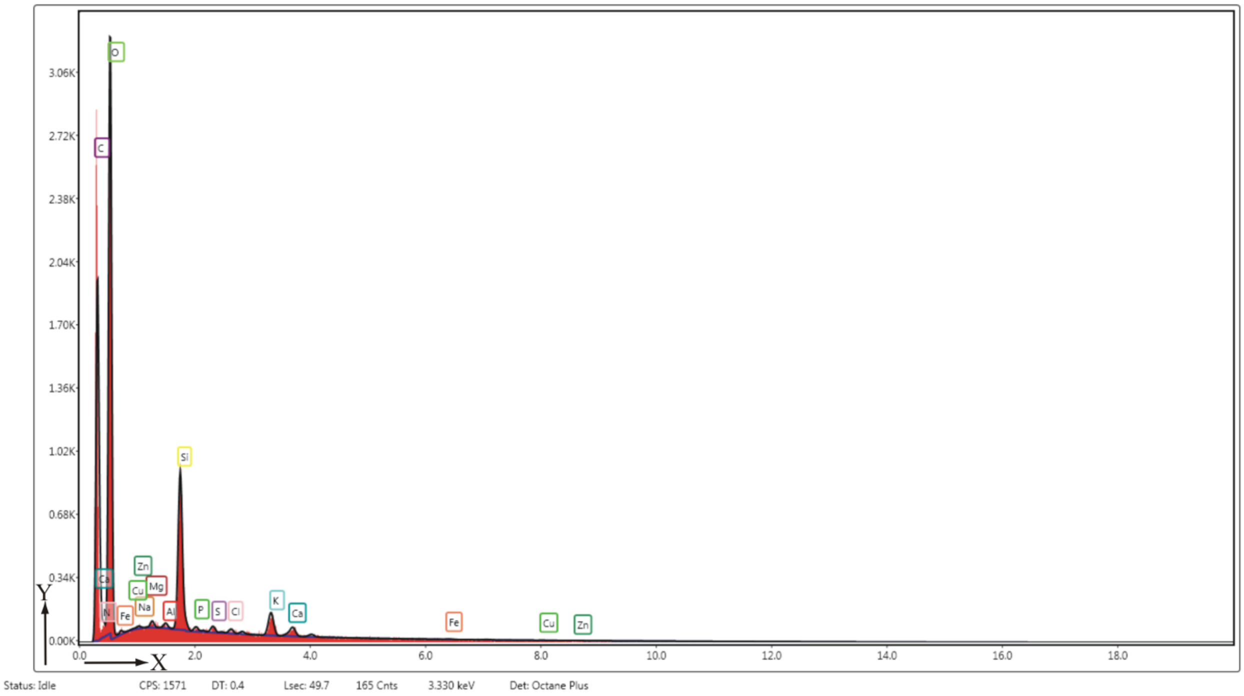

(b)

Legends

X axes: Energy (Kilo Electron Volt)

Y axes: Intensities

Supplementary Fig. 3. FESEM-EDS analysis micrographs in red pandas samples collected from summer (a) and winter (b).

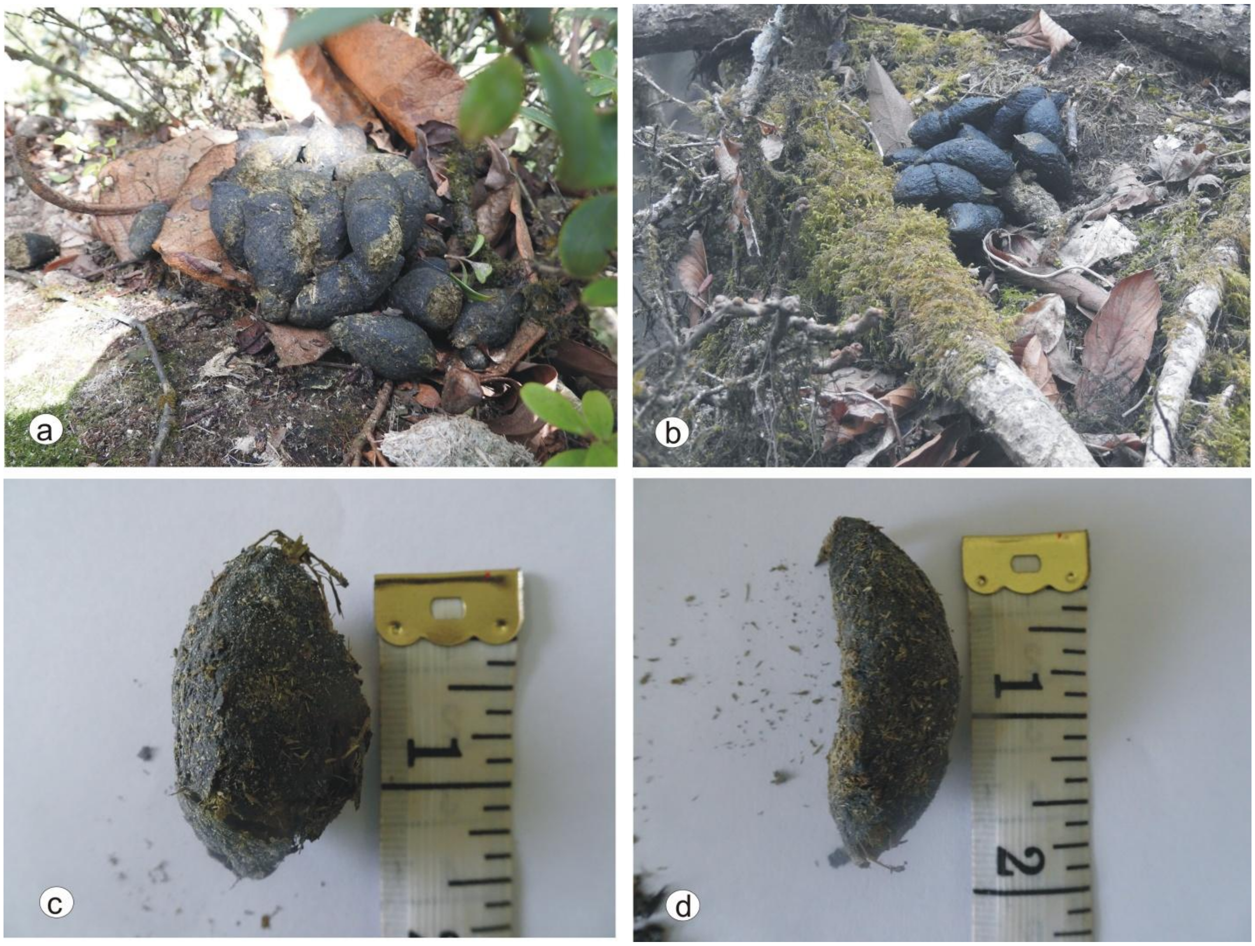

Supplementary Fig. 4. a and **b.** A feces midden of red panda, **c** and **d.** Size and shape of the feces.

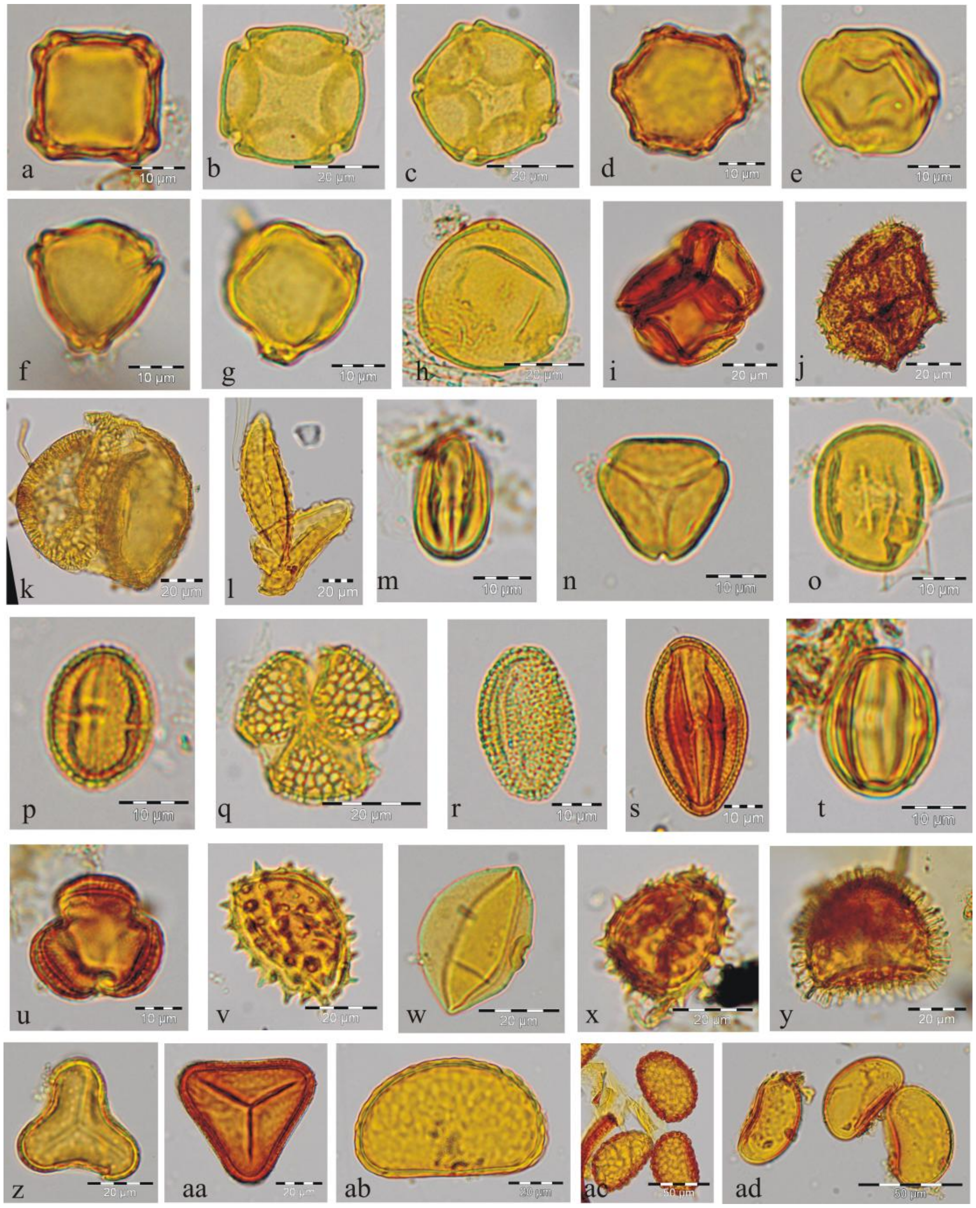

Supplementary Fig. 5. Pollen assemblage recovered from the *Ailurus fulgens* feces samples.

a. and **b.** *Alnus* sp., **c.** and **d.** *Corylus* sp., **e.** *Juglans*, **f.** and **g.** *Betula* sp., **h.** *Carya*, **i.** *Rhododendron* sp. **j.** *Rhododendron lecilatum*, **k.** *Pinus* sp., **l.** Arecaceae, **m.** *Castanopsis* sp., **n.** *Syzygium* sp., **o.** Meliaceae, **p.** *Olea* sp., **q.** *Ligustrum* sp., **r.** Oleaceae, **s.** Anacardiaceae, **t.** Combretaceae, **u.** *Artemisia* sp., **v.** Malvaceae, **w.** Bambusoideae, **x.** Asteroideae, **y.** *Lycopodium* sp., **z.** and **aa.** Trilete, **ab.** Monolete, **ac.** and **ad.** Clustering of monolete.

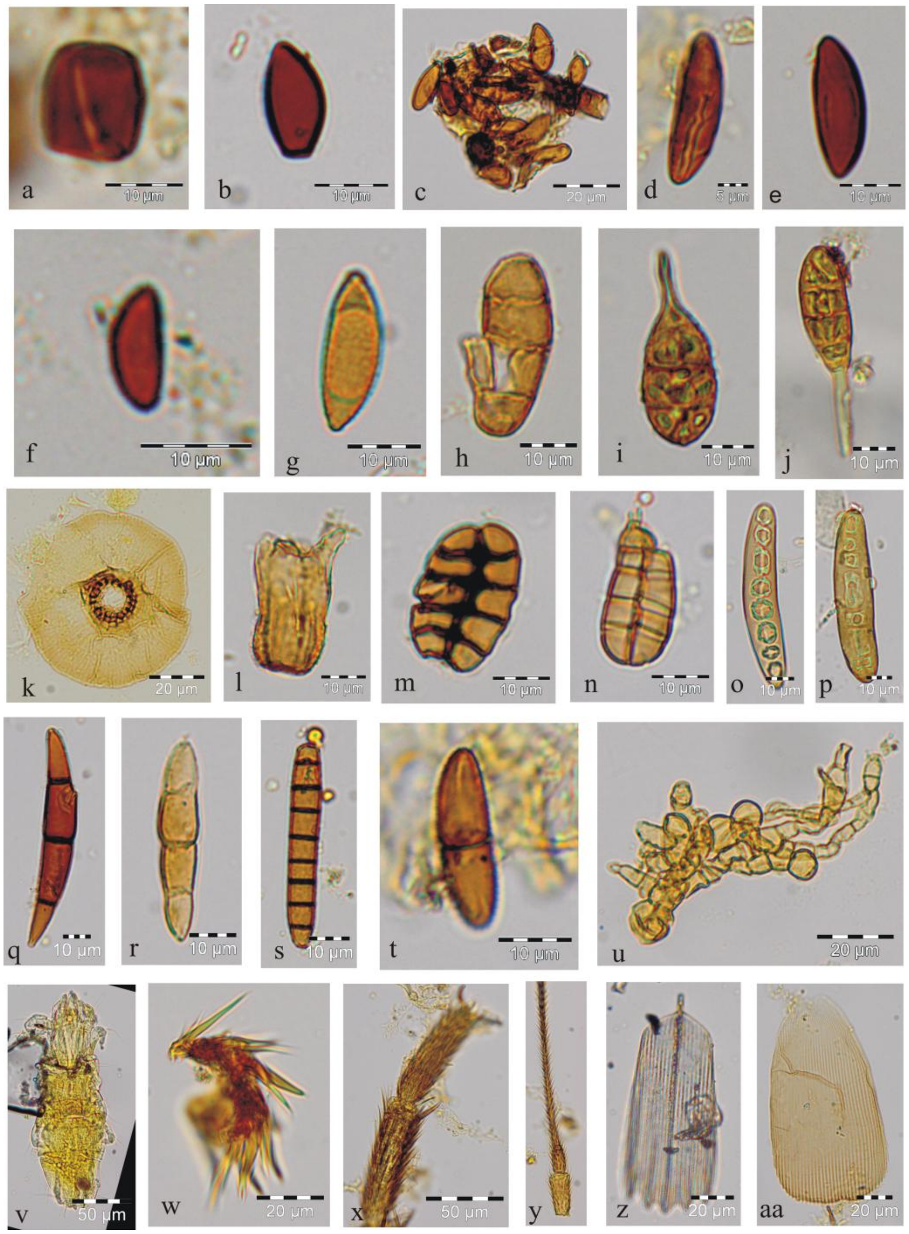

Supplementary Fig. 6. Non-pollen palynomorphs assemblage recovered from the red panda feces samples.

a. *Sporormiella*, **b.** *Cercophora*, **c.** Clustering of *Cercophora*, **d. - f.** *Podospora* sp., **g.** *Melanospora*, h. *Meliola*, i. and j. *Alternaria* sp., **k.** Microthyriaceae, l. *Tetraploa* sp., **m.** *Canalisporium* sp., n. *Pleospora* sp., o. and, p *Bipolaris* sp., **q.** and **r.** *Curvularia* sp., s. *Geoglossum* sp., **t.** *Arnium*, **u.** Clustering of mycelium, **v.** Acari, **w. -aa.** Insect body parts.

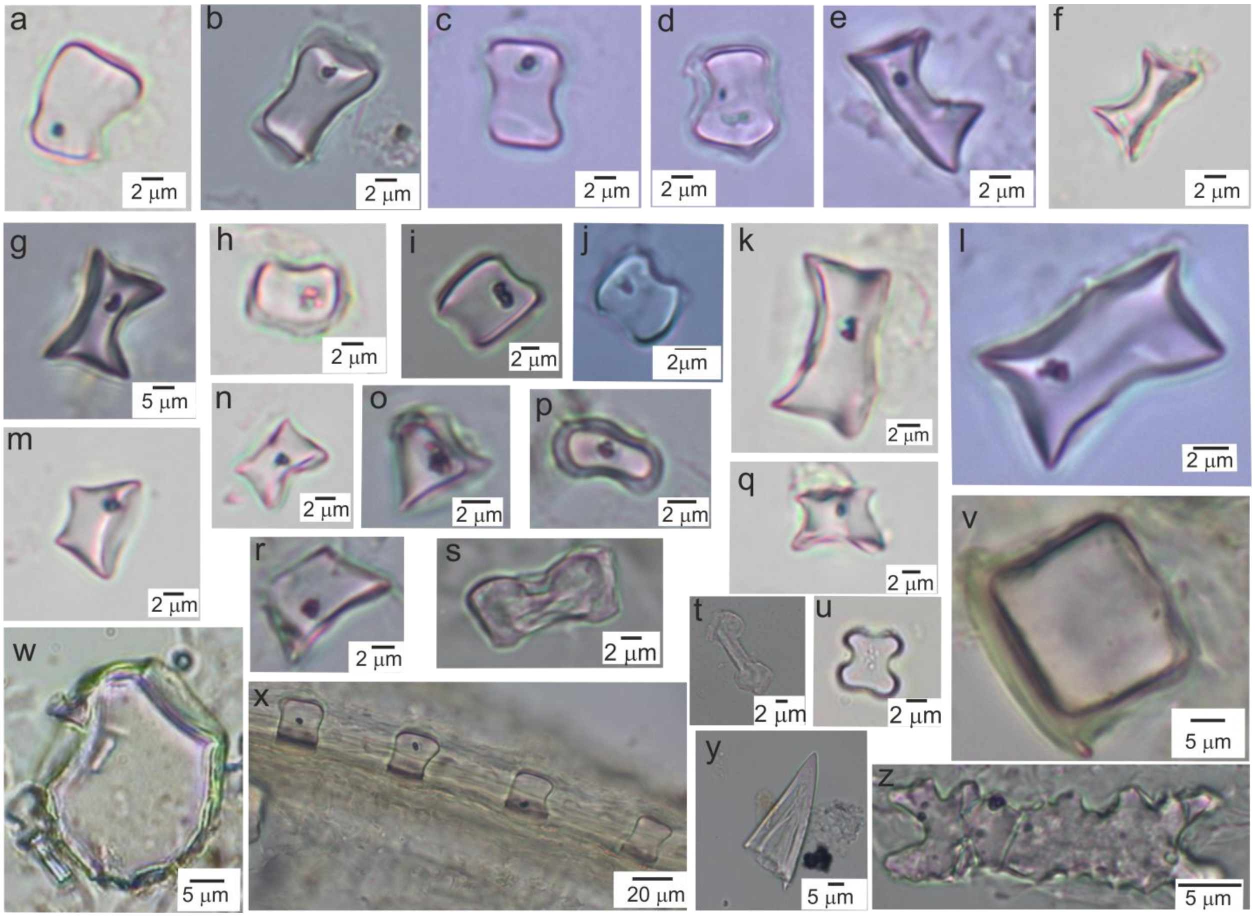

Supplementary Fig. 7. Some dominant phytolith morphotypes recovered from feces samples of red panda.

a–d. saddle tall, **e–g.** saddle collapsed, **h–j.** saddle, **k–l.** trapezoid, **m.** rondel two-horned, **n.** tower spool/horned, **o.** rondel pyramidal, **p.** rondel oval, **q.** rondel wavy top, **r.** tower flat, **s–t.** bilobate, **u.** cross, **v.** blocky, **w.** bulliform flabellate, **x.** saddle tall types within grass cuticle, **y.** acute bulbosus, **z.** elongate sinuate.

Supplementary Table 1. Summary results of detrended correspondence analysis (DCA) and principal component analysis (PCA) on the phytolith dataset from the summer and winter feces. Here, SD = standard deviation units for DCA.

**Pollen data**	DCA			
	Axis 1	Axis 2	Axis 3	Axis 4
Eigenvalue	0.096	0.001	0.001	0.000
Gradient length (SD)	0.657	0.118	0.170	0.087
Cumulative percentage variance of phytolith data (%)	93.1	94.1	94.8	95.0

	PCA			
	Axis 1	Axis 2	Axis 3	Axis 4
Eigenvalue	0.890	0.043	0.014	0.014
Cumulative percentage variance of species data (%)	89.0	93.3	94.7	96.1

**NPP data**	DCA			
	Axis 1	Axis 2	Axis 3	Axis 4
Eigenvalue	0.013	0.002	0.001	0.000
Gradient length (SD)	0.351	0.307	0.235	0.251
Cumulative percentage variance of phytolith data (%)	46.4	54.2	57.0	58.7

	PCA			
	Axis 1	Axis 2	Axis 3	Axis 4
Eigenvalue	0.559	0.143	0.086	0.063
Cumulative percentage variance of species data (%)	55.9	70.2	78.8	85.1

**Phytolith data**	DCA			
	Axis 1	Axis 2	Axis 3	Axis 4
Eigenvalue	0.156	0.098	0.046	0.030

Gradient length (SD)	1.520	1.165	0.978	0.899
Cumulative percentage variance of phytolith data (%)	20.9	34.0	40.2	44.2

	PCA			
	Axis 1	Axis 2	Axis 3	Axis 4
Eigenvalue	0.220	0.180	0.132	0.104
Cumulative percentage variance of species data (%)	22.0	40.0	53.2	63.6

Supplementary Table 2. Grass phytoliths recovered from the fecal matters of red panda collected during the summer and winter seasons and their abbreviations (Naming, coding and anatomical origin of the morphotypes are made following ICPN 2.0, ICPT: Neumann et al., 2019).

**Morphotypes**	**Acronyms**	**Anatomical origin**
Bilobate	BIL	Epidermis, and the side (long axis) of the morph is parallel with the long axis of the leaf
Cross	CRO	Epidermis, and its side is parallel or perpendicular to the long axis of the leaf
Saddle	SAD	Epidermis, and its side is parallel with the long axis of the leaf
Polylobate	POL	Epidermis, and the side is parallel to the long axis of the leaf
Saddle tall	SAD_TAL	Epidermis, and its side is parallel with the long axis of the leaf
Saddle collapsed	SAD_COL	Epidermis, and side is parallel with the long axis of the leaf
Rondel two-horned	RON_TWO	Epidermis, and its side is perpendicular to the long axis of the leaf
Rondelkeeled	RON_KEE	Epidermis, and its side is perpendicular to the long axis of the leaf
Rondelpyramidal	RON_PYR	Epidermis, and its side is perpendicular to the long axis of the leaf
Rondel oval	RON_OVA	Epidermis, and its side is perpendicular to the long axis of the leaf

Rondel wavy top	RON_WAV	Epidermis, and its side is perpendicular to the long axis of the leaf
Tower spool/horned	TOW_SPO	Epidermis, and the side of the morph is parallel with the long axis of the leaf
Tower flat	TOW_FLA	Epidermis, and the side of the morph is parallel with the long axis of the leaf
Trapezoid	TRZ	Epidermis, and side is parallel with the long axis of the leaf
Acute bulbosus	ACU_BUL	Silicification of the cell lumen of a hair cell
Blocky	BLO	Sub-epidermal cell of leaf
Bulliform flabellate	BUL_FLA	Specialised epidermal cell of leaf
Elongate entire	ELO_ENT	Leaf epidermis (long cell)
Elongate sinuate	ELO_SIN	Leaf epidermis (long cell)
Epidermis	EPI	Interior cast of epidermal cell
Hair base	HAI	Silicification of the cell lumen of a hair cell
Stomate	STO	Interior cast of stomata

Supplementary Table 3. List of the elements value generated by FESEM-EDS analysis in red panda feces sample collected from summer season.

Element	Weight %	Atomic %	Error %	Net Int.	K Ratio	Z	R	A	F
C K	35.23	42.61	99.99	239.47	0.1559	1.0326	0.9847	0.4285	1
N K	5.4	5.6	99.99	12.17	0.0075	1.0082	0.9948	0.1373	1
O K	54.41	49.42	9.52	422.44	0.1152	0.987	1.0039	0.2145	1
Na K	0	0	99.99	0.02	0	0.8954	1.027	0.3369	1.0022
Mg K	0.17	0.1	39.74	5.27	0.0008	0.9104	1.0335	0.5067	1.0039
Al K	0.11	0.06	63.76	4.2	0.0006	0.8764	1.0396	0.6667	1.0067
Si K	3.12	1.62	4.74	147.71	0.0223	0.8954	1.0454	0.7943	1.0063
P K	0.11	0.05	42.78	4.62	0.0008	0.8598	1.0508	0.8529	1.0099
S K	0.15	0.07	30.15	6.56	0.0012	0.8764	1.0559	0.9194	1.0141
Cl K	0.12	0.05	42.14	4.86	0.001	0.8336	1.0606	0.9632	1.021
K K	0.76	0.28	10.43	26.96	0.0066	0.8292	1.0692	1.0092	1.0346
Ca K	0.37	0.13	22.66	11.15	0.0033	0.8442	1.073	1.0156	1.0416
Fe K	0.04	0.01	65.35	0.67	0.0004	0.7519	1.088	1.023	1.2189
Cu K	0.01	0	83.96	0.17	0.0002	0.7192	1.0872	1.0182	1.4311
Zn K	0	0	97.04	0.02	0	0.7164	1.085	1.017	1.5269

Supplementary Table 4. List of the elements value generated by FESEM-EDS analysis in red panda feces sample collected from winter season.

Element	Weight %	Atomic %	Error %	Net Int.	K Ratio	Z	R	A	F
C K	35.93	43.23	99.99	247.82	0.1634	1.0316	0.9853	0.4409	1
N K	6.13	6.32	99.99	13.55	0.0084	1.0071	0.9954	0.1367	1
O K	53.53	48.35	9.6	399.66	0.1104	0.986	1.0044	0.2092	1
Na K	0	0	99.99	0.02	0	0.8944	1.0274	0.338	1.0022
Mg K	0.13	0.08	65	4.02	0.0006	0.9094	1.0339	0.5081	1.0037
Al K	0.07	0.04	65.87	2.71	0.0004	0.8754	1.0401	0.6686	1.0065
Si K	2.85	1.47	4.78	133.36	0.0204	0.8944	1.0458	0.7963	1.0063
P K	0.1	0.05	54.91	4.16	0.0008	0.8588	1.0512	0.8574	1.0098
S K	0.12	0.06	35.37	5.37	0.001	0.8755	1.0562	0.9231	1.0141
Cl K	0.1	0.04	57.96	4.09	0.0008	0.8327	1.061	0.9662	1.0209
K K	0.6	0.22	13.1	21.2	0.0052	0.8283	1.0695	1.011	1.0357
Ca K	0.35	0.13	21.98	10.57	0.0031	0.8433	1.0733	1.0177	1.043
Fe K	0.04	0.01	65.91	0.6	0.0003	0.751	1.0882	1.0234	1.227
Cu K	0.03	0.01	79.91	0.3	0.0003	0.7183	1.0874	1.0189	1.4441
Zn K	0.01	0	86.97	0.07	0.0001	0.7156	1.0851	1.017	1.5415

Supplementary Table 5. List of the main pollen taxa recovered from the red panda feces samples and their flowering period.

**Serial no**	**Major plant taxa recovered in pollen assemblage**	**Flowering period**
1	*Betula* (Betulaceae)	January to June
2	*Alnus* (Betulaceae)	July to October
3	*Corylus* (Betulaceae)	November to December
4	*Juglans* (Juglandaceae)	April to May
5	*Quercus* (Fagaceae)	March to July
6	*Rhododendron* (Ericaceae)	March to September
7	*Syzygium* (Myrtaceae)	November to July
8	Meliaceae	February to June

9	Combretaceae	March to August
10	Oleaceae	February to July
11	*Ligustrum* (Oleaceae)	April to July
12	Anacardiaceae	March to July
13	Bambosoideae	July to October
14	*Artemisia* (Asteraceae)	July to October
15	Ferns (spore formation)	July to October
